# Smoking Cessation Pharmacotherapy Use in Pregnancy

**DOI:** 10.1001/jamanetworkopen.2024.19245

**Published:** 2024-06-28

**Authors:** Annelies L. Robijn, Duong T. Tran, Jacqueline M. Cohen, Sarah Donald, Carolyn E. Cesta, Kari Furu, Lianne Parkin, Sallie-Anne Pearson, Johan Reutfors, Helga Zoega, Nicholas Zwar, Alys Havard

**Affiliations:** 1 National Drug and Alcohol Research Centre, Faculty of Medicine and Health, UNSW Sydney, Sydney, New South Wales, Australia; 2 Department of Chronic Diseases & Centre for Fertility and Health, Norwegian Institute of Public Health, Oslo, Norway; 3 Department of Preventive and Social Medicine, Dunedin School of Medicine, University of Otago, Dunedin, New Zealand; 4 Centre for Pharmacoepidemiology, Department of Medicine Solna, Karolinska Institutet, Stockholm, Sweden; 5 Medicine Intelligence Research Program, School of Population Health, Faculty of Medicine and Health, UNSW Sydney, Sydney, New South Wales, Australia; 6 Centre of Public Health Sciences, Faculty of Medicine, University of Iceland, Reykjavik, Iceland; 7 Faculty of Health Sciences & Medicine, Bond University, Gold Coast, Queensland, Australia

## Abstract

**Importance:**

Significant evidence gaps exist regarding the safety of smoking cessation pharmacotherapies during pregnancy, especially for the risk of congenital malformations. Consequently, professional bodies advise against the use of varenicline and bupropion and recommend caution with nicotine replacement therapy (NRT). Contemporary estimates of the use of smoking cessation pharmacotherapies during pregnancy are lacking.

**Objective:**

To quantify the proportion of individuals using prescribed smoking cessation pharmacotherapies during pregnancy and during the first trimester specifically, in 4 countries.

**Design, Setting, and Participants:**

This retrospective, population-based cohort study used linked birth records, hospital admission records, and dispensing records of prescribed medications from all pregnancies resulting in birth between 2015 and 2020 in New South Wales, Australia; New Zealand; Norway; and Sweden. Data analyses were conducted in October and November 2023.

**Exposure:**

Prescribed smoking cessation pharmacotherapy use (varenicline, NRT, and bupropion) during pregnancy was defined as days’ supply overlapping the period from date of conception to childbirth.

**Main Outcomes and Measures:**

Prevalence of use among all pregnancies and pregnancies with maternal smoking were calculated. Among women who used a pharmacotherapy, the proportion of women with use during the first trimester of pregnancy was also calculated.

**Results:**

Among 1 700 638 pregnancies in 4 countries, 138 033 (8.1%) had maternal smoking and 729 498 (42.9%) were younger than 30 years. The prevalences ranged from 0.02% to 0.14% for varenicline, less than 0.01% to 1.86% for prescribed NRT, and less than 0.01% to 0.07% for bupropion. Among pregnant individuals who smoked, use of pharmacotherapies was up to 10 times higher, with maximum prevalences of 1.25% for varenicline in New South Wales, 11.39% for NRT in New Zealand, and 0.39% for bupropion in New Zealand. Use in the first trimester occurred among more than 90% of individuals using varenicline, approximately 60% among those using NRT, and 80% to 90% among those using bupropion.

**Conclusions and Relevance:**

In this cohort study of pregnant individuals in 4 high-income countries, the low prevalence of varenicline and bupropion use during pregnancy and higher prevalence of NRT use aligned with current clinical guidelines. As most use occurred in the first trimester, there is a need for evidence on the risk of congenital malformations for these medications.

## Key Points

**Question:**

How many pregnancies include use of prescription smoking cessation pharmacotherapies, especially during the first trimester?

**Findings:**

In this cohort study of 1.7 million pregnancies in New South Wales, Australia; New Zealand; Norway; and Sweden from 2015 to 2020, use of varenicline and bupropion during pregnancy was low, while prescription nicotine replacement therapy was used during a larger proportion of pregnancies. For most pregnancies in which these medications were used, use occurred at least partly during the first trimester.

**Meaning:**

These findings suggest that maternal use of smoking cessation pharmacotherapies aligns with current clinical recommendations; however, robust evidence on safety during pregnancy is needed.

## Teaser

This cohort study describes the proportion and characteristics of individuals in 4 high-income countries using prescribed smoking cessation pharmacotherapy during pregnancy overall and especially during the first trimester.

## Introduction

Maternal smoking during pregnancy is a leading, yet modifiable, cause of adverse pregnancy and childhood outcomes.^1,2^ The prevalence of smoking during pregnancy is declining in high-income countries; nevertheless, most contemporary estimates indicate 6% to 18% of pregnant individuals smoke.^3,4,5^ Although the risk of adverse outcomes can be significantly reduced by quitting in the first half of pregnancy, less than 50% of individuals who smoke succeed with this.^6^ For pregnant individuals, current first-line quit-smoking options include counselling, peer support, and feedback, but the effectiveness of these behavioral interventions is modest.^7,8,9^ Smoking cessation pharmacotherapies, including varenicline, nicotine replacement therapy (NRT), and bupropion, are considered the most effective smoking cessation strategy in the general population.^10^ Although the evidence around the efficacy and effectiveness of these medications during pregnancy is not definitive,^11,12^ they may be a promising option for smoking cessation in pregnancy.

Despite encouraging findings, there is no conclusive evidence regarding the safety of smoking cessation pharmacotherapies when used during pregnancy, especially regarding congenital malformations.^11,13^ Consequently, many professional bodies advise against the use of varenicline and bupropion during pregnancy, indicating that NRT can be used during pregnancy if a pregnant individual is unable to quit with behavioral support alone.^14,15,16,17,18,19^ Despite the potential risks, it is unknown how many individuals use these medications during pregnancy and whether use is consistent with guidelines. Prior studies^20,21,22,23,24,25^ have generally been conducted with data from more than 10 years ago and report that among individuals who smoked during pregnancy, between 2.3% and 11.1%^21,22,23,25^ used a smoking cessation pharmacotherapy. Among all pregnant individuals, regardless of smoking status, approximately 2.4% used any pharmacotherapy^20^ and 2% used prescription NRT.^24^ Despite evaluation over the last decade, no strong evidence of risk has emerged for these products,^11,26,27,28^ which may change perceptions among pregnant individuals and health care practitioners toward using these therapies in pregnancy. The increased use of electronic cigarettes may also have contributed to changes in the uptake of pharmacotherapies. It is therefore important to determine prevalence of smoking cessation pharmacotherapy use during pregnancy using recent data and to specifically quantify use in the first trimester, as this is when the fetus is at risk of developing congenital malformations.

In this population-based study, we examined the use of smoking cessation pharmacotherapies in 4 countries from 2015 to 2020, among all pregnancies and among those with maternal smoking. Specifically, we leveraged population-based registry data to determine the proportion of pregnancies resulting in birth during which women used any smoking cessation pharmacotherapy, specific pharmacotherapies (NRT, varenicline, and bupropion), and specific NRT formulations. It is important to understand use of different formulations, as the mode of nicotine delivery influences the total daily dose received and animal studies have suggested that some of the adverse outcomes of nicotine use during pregnancy are dose dependent.^29^ We further determined use during the first trimester among all pregnancies, due to safety concerns regarding congenital malformations.

## Methods

This cohort study was approved by the Australian Institute of Health and Welfare Human Research Ethics Committee, the New South Wales Population and Health Services Research Ethics Committee, the University of Otago Human Ethics Committee (Health), the Norwegian Regional Ethics Committee, and the Swedish ethics committee. A waiver of the requirement for informed consent was granted by a relevant ethics committee in each jurisdiction owing to the impracticability to obtain consent for this whole-of-population research, and a waiver was granted on the basis that the benefits of the research outweighed the risks of harm associated with not seeking consent. This study is reported following the Strengthening the Reporting of Observational Studies in Epidemiology (STROBE) reporting guideline and the Reporting of Studies Conducted Using Observational Routinely Collected Health Data Statement for Pharmacoepidemiology (RECORD-PE).

### Study Design and Data Sources

We conducted a population-based study in 4 countries, including New South Wales, the most populous state in Australia; New Zealand; Norway; and Sweden. Data were linked at the person level and included perinatal or birth registers, hospital admission records or specialist health care registers, and records of dispensed prescribed medications. A summary of these data collections can be found in eTable 1 in Supplement 1.

### Study Population

The study population included all pregnancies resulting in birth (gestational age ≥22 weeks), both live birth and stillbirth, between January 2015 and December 2019 in New South Wales and Sweden and between January 2015 and December 2020 in New Zealand and Norway. We excluded pregnancies with missing or invalid gestational age (≥44 weeks) or in which the mother was an overseas visitor (New South Wales and New Zealand) or immigrated into the country during pregnancy (Norway and Sweden).

### Smoking Cessation Pharmacotherapy Use

We identified smoking cessation pharmacotherapies from prescription medication dispensing databases. In all included countries, varenicline is available by prescription only. Bupropion is also prescription only; however, in New South Wales and New Zealand^30^ bupropion is only approved as a smoking cessation aid, while in Norway and Sweden it can be prescribed for smoking cessation or depression treatment. When analyzing the Norwegian and Swedish data, we included only sustained-release bupropion (sold under brand name Zyban), as this is indicated for smoking cessation exclusively. Although NRT can be purchased over-the-counter in all participating countries, in this study, we only had data relating to prescribed NRT. Differences in reimbursement policies for prescribed NRT across the participating countries affects the extent to which prescribed NRT use can be considered indicative of total NRT use. Further details on smoking cessation pharmacotherapy availability in each country are provided in eTable 2 in Supplement 1.

We identified use of a smoking cessation pharmacotherapy during pregnancy as when a course of treatment overlapped with the gestation period (ie, from date of conception to date of childbirth) (eTable 2 and eFigure 1 in Supplement 1). Date of conception (DOC) was estimated using the formula date of childbirth − gestational age in weeks × 7 + 14 days.

Timing of use was categorized in 6 mutually exclusive categories based on timing of the first dispensing of a course (Table 1). The first trimester was defined as the period from DOC to DOC + 83 days, while the second and third trimester was defined as the period from DOC + 84 to date of childbirth. Pregnancies with first trimester use were subdivided based on whether the use commenced in the preconception period (DOC − 200 days until DOC − 1 day) or in the first trimester. We made this distinction in recognition of the possibility that individuals may stop using medications on recognition of their pregnancy. To gauge the likelihood of use of the dispensed product, we further subcategorized each use category based on whether the mother received a subsequent dispensing of the same therapy within a course of treatment.

**Table 1.** Timing of Use Categories

Category	First dispensing of course occurring	No. of dispensings
Preconception and pregnancy use, including first trimester	Preconception (supply of course overlaps with conception date)	1
		≥2^a^
Pregnancy use, including first trimester	First trimester	1
		≥2
Pregnancy use, excluding first trimester	Second or third trimester	1
		≥2

^a^ At least 1 subsequent dispensing in the course occurs after date of conception and after the date of the first dispensing.

### Maternal Smoking Status

An aim of this study was to determine smoking cessation pharmacotherapy use among pregnancies with maternal smoking (in addition to all pregnancies). We identified maternal smoking status based on information indicating the smoking status and quantity smoked that was recorded in the perinatal or medical birth records, supplemented with the presence of the *International Statistical Classification of Diseases and Related Health Problems, Tenth Revision (ICD-10)* code Z72.0 (Tobacco use, current) in the hospital stay relating to birth.^31^ Pregnancies with a smoking indicator in either the perinatal or inpatient record were classified as belonging to individuals with a record of smoking. Acknowledging the underrecording of smoking during pregnancy, we used a published algorithm^22^ to identify additional pregnancies during which individuals smoked. This algorithm analyses patterns of smoking pharmacotherapy dispensings (eFigure 2 and eTable 3 in Supplement 1). We refer to additional pregnancies identified through this algorithm as belonging to individuals with a reclassified smoking status.

### Maternal Characteristics

We included the following characteristics to describe the populations in each country: year of childbirth, mother’s sociodemographic characteristics, obstetric history, morbidities, smoking in early pregnancy, and quantity smoked in early pregnancy. In Australia and New Zealand, population characteristics also included registry data on indigenous status (ie, Aboriginal and/or Torres Strait Islander in Australia and Māori in New Zealand). Indigenous status was included in the descriptive statistics because smoking prevalence is substantially higher in Aboriginal and Torres Strait Islander individuals and Maori individuals; therefore, understanding their representation in the study population is important for interpreting the utilization rates. These variables were derived from information in perinatal, maternity, or medical birth register data, inpatient data, and medication dispensing data. Detailed definitions of characteristics and maternal morbidities are listed in eTable 4 and eTable 5 in Supplement 1.

### Statistical Analysis

Data analyses were conducted in October and November 2023. To adhere to privacy laws, we analyzed unit record data within each country and shared summarized data using a common protocol. We used descriptive statistics (frequency and percentage) to describe characteristics of the study population. In each country, we calculated the prevalence of smoking cessation pharmacotherapy use as any pharmacotherapy and for each pharmacotherapy separately. We estimated prevalence of use among all pregnancies resulting in birth, and among pregnancies with maternal smoking. We present prevalences among pregnancies with maternal smoking as a range, with the lower bound representing prevalence of use among pregnancies with a record of smoking and the upper bound representing prevalence of use among pregnancies with a record of smoking and pregnancies with a reclassified smoking status. We calculated the proportion of pregnancies with pharmacotherapy use according to the timing of use categories. We summarized how many individuals had the medication in their possession during the first trimester by summing the 4 categories with first trimester use. Analyses were conducted in SAS software version 9.4 (SAS Institute) for data from New South Wales and Stata software version 17 (StataCorp) for data from New Zealand, Norway, and Sweden.

To facilitate comparisons with studies in which the last menstrual period (LMP) date is considered the start date of pregnancy, we conducted a sensitivity analysis in which we recalculated the prevalence of use. We defined use of a smoking cessation pharmacotherapy during pregnancy if a course overlapped with any day between LMP and date of childbirth.

## Results

In total, we identified 1 725 803 pregnancies resulting in birth in the study period in the 4 countries. After exclusions, 1 700 638 pregnancies were included in the study population (eFigure 3 in Supplement 1), including 729 498 among individuals younger than 30 years and among 118 690 (7.0%) with smoking in early pregnancy. Smoking in early pregnancy (during first 20 weeks in New South Wales, at first lead maternity carer visit in New Zealand, during first trimester in Norway, and at first antenatal visit in Sweden) was recorded most often in New Zealand (44 951 of 352 412 pregnancies [12.8%]) and least often in Norway (11 161 of 324 926 pregnancies [3.4%]). Table 2 describes the characteristics of the pregnancies according to smoking cessation pharmacotherapy use. In New South Wales and New Zealand, individuals using a pharmacotherapy tended to be younger, whereas individuals using a pharmacotherapy in Norway and Sweden tended to be older (Table 2). Individuals who used a pharmacotherapy during pregnancy often had previous births and were more likely to have a health condition, particularly mental health disorders, compared with individuals who did not use a pharmacotherapy (Table 2).

**Table 2.** Characteristics of the Study Population, by Country and Use and Nonuse of Any Smoking Cessation Pharmacotherapy During Pregnancy

Characteristic	Pregnancies by smoking cessation pharmacotherapy use, No. (%)							
	New South Wales, Australia		New Zealand		Norway		Sweden	
	Use (n = 1728)	Nonuse (n = 466 613)	Use (n = 7100)	Nonuse (n = 345 312)	Use (n = 77)	Nonuse (n = 324 849)	Use (n = 261)	Nonuse (n = 554 698)
Year of childbirth								
2015	310 (17.9)	93 199 (20.0)	1553 (21.9)	56 860 (16.5)	18 (23.4)	56 838 (17.5)	52 (19.9)	109 953 (19.8)
2016	309 (17.9)	96 027 (20.6)	1372 (19.3)	57 912 (16.8)	13 (16.9)	56 620 (17.4)	42 (16.1)	112 505 (20.3)
2017	375 (21.7)	93 295 (20.0)	1236 (17.4)	57 982 (16.8)	13 (16.9)	54 255 (16.7)	57 (21.8)	110 287 (19.9)
2018	378 (21.9)	92 293 (19.8)	1000 (14.1)	57 039 (16.5)	15 (19.5)	53 083 (16.3)	59 (22.6)	111 488 (20.1)
2019	356 (20.6)	91 799 (19.7)	1000 (14.1)	58 327 (16.9)	6 (7.8)	52 632 (16.2)	51 (19.5)	110 465 (19.9)
2020	NA	NA	939 (13.2)	57 192 (16.6)	12 (15.6)	51 421 (15.8)	NA	NA
Age, y								
<25	402 (23.3)	61 304 (13.1)	1930 (27.2)	65 549 (19.0)	5 (6.5)	34 492 (10.6)	31 (11.9)	63 201 (11.4)
25-29	481 (27.8)	122 753 (26.3)	2167 (30.5)	94 953 (27.5)	28 (36.4)	104 097 (32.0)	68 (26.1)	178 127 (32.1)
30-34	486 (28.1)	167 654 (35.9)	1793 (25.3)	111 153 (32.2)	21 (27.3)	117 714 (36.2)	90 (34.5)	190 374 (34.3)
≥35	359 (20.8)	114 820 (24.6)	1210 (17.0)	73 657 (21.3)	23 (29.9)	68 546 (21.1)	72 (27.6)	122 996 (22.2)^a^
Unknown or missing	NA	82 (<0.1)	NA	NA	NA	NA	NA	NA
BMI								
<18.5	78 (4.5)	16 623 (3.6)	170 (2.4)	8413 (2.4)	NA	NA	5 (1.9)	13 116 (2.4)
18.5-24.9	585 (33.9)	193 062 (41.4)	2186 (30.8)	139 419 (40.4)	23 (29.9)^b^	180 202 (55.5)^b^	87 (33.3)	294 885 (53.2)
25-29.9	377 (21.8)	86 515 (18.5)	2017 (28.4)	93 088 (27.0)	19 (24.7)	63 023 (19.4)	81 (31.0)	137 949 (24.9)
≥30	376 (21.8)	65 175 (14.0)	2343 (33.0)	88 685 (25.7)	24 (31.2)	35 753 (11.0)	72 (27.6)	77 216 (13.9)
Unknown or missing	312 (18.1)	105 238 (22.6)	384 (5.4)	15 707 (4.5)	11 (14.3)	45 871 (14.1)	16 (6.1)	31 532 (5.7)
Recorded smoking in early pregnancy^c^								
Yes	1102 (63.8)	37 744 (8.1)	4492 (63.3)	40 459 (11.7)	38 (49.4)	11 123 (3.4)	97 (37.2)	23 635 (4.3)
No	618 (35.8)	427 577 (91.6)	2239 (31.5)	289 884 (83.9)	31 (40.3)	285 127 (87.8)	160 (61.3)	514 841 (92.8)
Unknown or missing	8 (0.5)	1292 (0.3)	369 (5.2)	14 969 (4.3)	8 (10.4)	28 599 (8.8)	4 (1.5)	16 222 (2.9)
Quantity smoked among those with recorded smoking, cigarettes/d								
<10	818 (74.2)	31 807 (84.3)	3008 (67.0)^d^	29 552 (73.0)	12 (31.6)	5325 (47.9)	66 (68.0)	18 927 (80.1)
≥10	284 (25.8)	5927 (15.7)	1484 (33.0)	10 895 (26.9)	22 (57.9)	3201 (28.8)	31 (32.0)	4708 (19.9)
Unknown or missing	NA	10 (<0.1)	NA	12 (<0.1)	4 (10.5)	2597 (23.3)	NA	NA
Country of birth								
Same as country of delivery	1580 (91.4)	291 187 (62.4)	NA	NA	62 (80.5)	233 434 (71.9)	195 (74.7)	399 896 (72.1)
Other	148 (8.6)	175 094 (37.5)	NA	NA	15 (19.5)	89 832 (27.7)	66 (25.3)	154 599 (27.9)
Unknown or missing	NA	332 (0.2)	NA	NA	NA	1583 (0.5)	NA	203 (<0.1)
Indigenous status^e^								
Yes	386 (22)	21 879 (5)	3636 (51.2)	94 827 (27.5)	NA	NA	NA	NA
No	1342 (78)	443 897 (95)	3459 (48.7)	250 374 (72.5)	NA	NA	NA	NA
Unknown or missing	NA	837 (<1)	5 (0.1)	111 (<0.1)	NA	NA	NA	NA
Socioeconomic disadvantage, quintile^f^								
1 (Most disadvantaged)	580 (33.6)	101 095 (21.7)	2437 (34.3)	94 564 (27.4)	NA	NA	NA	NA
2	544 (31.5)	96 405 (20.7)	1861 (26.2)	78 996 (22.9)	NA	NA	NA	NA
3	309 (17.9)	90 927 (19.5)	1296 (18.3)	62 717 (18.2)	NA	NA	NA	NA
4	167 (9.7)	71 199 (15.3)	916 (12.9)	57 711 (16.7)	NA	NA	NA	NA
5 (Least disadvantaged)	110 (6.4)	97 452 (20.9)	584 (8.2)	50 921 (14.7)	NA	NA	NA	NA
Unknown or missing	18 (1.0)	9535 (2.0)	6 (0.1)	403 (0.1)	NA	NA	NA	NA
Remoteness of residence^g^								
Most urban areas	988 (57.2)	351931 (75.4)	3852 (54.3)	225 198 (65.2)	NA	NA	NA	NA
Regional areas	528 (30.6)	79247 (17.0)	1689 (23.8)	61 012 (17.7)	NA	NA	NA	NA
Rural and remote areas	194 (11.2)	25888 (5.5)	1553 (21.9)	58 707 (17.0)	NA	NA	NA	NA
Unknown or missing	18 (1.0)	9547 (2.0)	6 (<0.1)	395 (<0.1)	NA	NA	NA	NA
Parity								
Nulliparous	540 (31.3)	201 718 (43.2)	2668 (37.6)	140 122 (40.6)	27 (35.1)	136 098 (41.9)	119 (45.6)	235 884 (42.5)
Primiparous	485 (28.1)	162 364 (34.8)	3939 (55.5)	188 991 (54.7)	23 (29.9)	122 525 (37.7)	76 (29.1)	206 572 (37.2)
Multiparous	703 (40.7)^h^	102 410 (21.9)	493 (6.9)	16 199 (4.7)	27 (35.1)	66 226 (20.4)	66 (25.3)	112 242 (20.2)
Unknown or missing	NA	121 (<0.1)	NA	NA	NA	NA	NA	NA
Maternal morbidities								
Preexisting diabetes	37 (2.1)	4232 (0.9)	129 (1.8)	3989 (1.2)	<5	8085 (2.5)	13 (5.0)	8988 (1.6)
Preexisting hypertension	37 (2.1)	5861 (1.3)	124 (1.7)	3389 (1.0)	<5	4499 (1.4)	6 (2.3)	6340 (1.1)
Gastroesophageal reflux	194 (11.2)	25 183 (5.4)	611 (8.6)	19 261 (5.6)	18 (23.4)	26 676 (8.2)	71 (27.2)	46 495 (8.4)
Epilepsy	47 (2.7)	3154 (0.7)	274 (3.9)	4551 (1.3)	0	2185 (0.7)	5 (1.9)	3308 (0.6)
Mental health disorder	705 (40.8)	56 560 (12.1)	2211 (31.1)	40 314 (11.7)	31 (40.3)	47 687 (14.7)	134 (51.3)	68 419 (12.3)
Chronic airways disease	334 (19.3)	35 351 (7.6)	1384 (19.5)	42 628 (12.3)	15 (19.5)	32 689 (10.1)	78 (29.9)	63 249 (11.4)
Thyroid disorder	47 (2.7)	18 919 (4.1)	91 (1.3)	7116 (2.1)	5 (6.5)	15 477 (4.8)	30 (11.5)	47 663 (8.6)

Abbreviations: BMI, body mass index (calculated as weight in kilograms divided by height in meters squared); NA, variable not available.

^a^ Contains fewer than 5 individuals (the reporting limit for Norwegian and Swedish data) with unknown age.

^b^ Contains individuals with BMI less than 18.5.

^c^ Defined as smoking during first 20 weeks’ gestation for New South Wales, smoking at first lead maternity carer registration for New Zealand, smoking in first trimester for Norway, and smoking at first antenatal visit (8-12 weeks’ gestation) in Sweden.

^d^ Contains fewer than 3 individuals (the reporting limit for New Zealand data) with unknown quantity smoked.

^e^ Indigenous status included Aboriginal and/or Torres Strait Islander for New South Wales and Māori for New Zealand.

^f^ The New Zealand Deprivation Index quintiles have been reversed to accommodate reporting of this study. Quintile 1 corresponds with quintile 5 and quintile 5 corresponds with quintile 1 in the New Zealand Deprivation Index.

^g^ Remoteness of residence is available in New Zealand and New South Wales data and not available in Norwegian and Swedish data. Most urban areas include the categories urban 1 (New Zealand)or major cities (New South Wales); regional areas include the categories urban 2 (New Zealand) or inner regional (New South Wales); rural and remote areas include the categories rural 1, rural 2, and rural 3 (New Zealand) and outer regional, remote, and very remote (New South Wales).

^h^ Contains fewer than 6 individuals (the reporting limit for New South Wales data) with unknown parity.

Table 3 describes the prevalence of smoking cessation pharmacotherapy use among all pregnancies (ranging from 0.02% to 2.01%) and among pregnancies with maternal smoking. The prevalence of varenicline use among all pregnancies varied from 0.02% (65 pregnancies in Norway) to 0.14% (643 pregnancies in New South Wales), bupropion prevalence ranged from less than 0.01% (fewer than 5 pregnancies in Norway and 26 pregnancies in Sweden) to 0.07% (256 pregnancies in New Zealand), and prescription NRT prevalence ranged from less than 0.01% (11 pregnancies in Norway) to 1.86% (6551 pregnancies in New Zealand). In New Zealand, among all pregnancies with prescription NRT use, 4854 of 6551 individuals (74.1%) used patches, 2097 individuals (32.0%) used lozenges, and 2642 individuals (40.3%) used gum; more than 1 form of NRT was used in 2680 pregnancies (40.9%).

**Table 3.** Prevalence of Use of Smoking Cessation Pharmacotherapies During Pregnancy Among All Pregnancies Across Countries

Therapy	Pregnancies, No. (%)			
	New South Wales, Australia (n = 468 341)	New Zealand (n = 352 412)	Norway (n = 324 926)	Sweden (n = 554 959)
Any smoking cessation pharmacotherapy	1728 (0.37)	7100 (2.01)	77 (0.02)	261 (0.05)
Varenicline	643 (0.14)	383 (0.11)	65 (0.02)	192 (0.03)
Bupropion	43 (0.01)	256 (0.07)	<5^a^	26 (<0.01)
NRT				
Any	1062 (0.23)^b^	6551 (1.86)	11 (<0.01)	46 (0.01)^c^
Patches	NA	4854 (1.38)	NA	26 (<0.01)
Lozenges	NA	2097 (0.60)	NA	10 (<0.01)
Gums	NA	2642 (0.75)	NA	6 (<0.01)

Abbreviations: NA, not applicable; NRT, nicotine replacement therapy.

^a^ Fewer than 5 pregnancies is the reporting limit for Norwegian and Swedish data.

^b^ In New South Wales, NRT patches were available on subsidy throughout the whole study period, while subsidy for lozenges and gums commenced in late 2019. As this resulted in small cell sizes, no stratification by NRT formulation was performed.

^c^ In Sweden, NRT nasal sprays and inhalers were used by fewer than 5 individuals.

Table 4 describes the prevalence of smoking cessation pharmacotherapy use in pregnancies with maternal smoking (ranging from 0.33% to 12.25%). Varenicline was used in 0.26% (30 pregnancies in Norway) to 1.25% (538 pregnancies in NSW) of pregnancies, bupropion was used in 0.03% (7 pregnancies in Sweden) to 0.39% (218 pregnancies in New Zealand) of pregnancies, and prescription NRT was used among 0.06% (7 pregnancies in Norway) to 11.39% (6414 pregnancies in New Zealand) of pregnancies. The distribution of use across different NRT formulations was similar to that observed for all pregnancies.

**Table 4.** Prevalence of Use of Smoking Cessation Pharmacotherapies During Pregnancy With Maternal Smoking Across Countries

Therapy	Pregnancies, No. (%)^a^							
	New South Wales, Australia		New Zealand		Norway		Sweden	
	Lower bound (n = 42 782)	Upper bound (n = 43 198)	Lower bound (n = 54 597)	Upper bound (n = 56 292)	Lower bound (n = 11 497)	Upper bound (n = 11 530)	Lower bound (n = 26 919)	Upper bound (n = 27 013)
Any smoking cessation pharmacotherapy	1169 (2.73)	1585 (3.67)	5201 (9.53)	6896 (12.25)	38 (0.33)	71 (0.62)	116 (0.43)	210 (0.78)
Varenicline	316 (0.74)	538 (1.25)	230 (0.42)	354 (0.63)	30 (0.26)	59 (0.51)	79 (0.29)	152 (0.59)
Bupropion	27 (0.06)	41 (0.09)	121 (0.22)	218 (0.39)	<5^b^	<5^b^	7 (0.03)	15 (0.06)
Prescription NRT								
Any	842 (1.97)^c^	1026 (2.38)^c^	4925 (9.02)	6414 (11.39)	7 (0.06)	11 (0.10)	32 (0.12)^d^	46 (0.17)^d^
Patches	NA	NA	3754 (6.88)	4782 (8.49)	NA	NA	18 (0.07)	26 (0.10)
Lozenges	NA	NA	1635 (2.99)	2089 (3.71)	NA	NA	<5^b^	6 (0.02)
Gums	NA	NA	1951 (3.57)	2575 (4.57)	NA	NA	8 (0.03)	10 (0.04)

Abbreviations: NA, not applicable; NRT, nicotine replacement therapy.

^a^ Lower bound represents use among pregnancies in which smoking was recorded in pregnancy, and upper bound represents use among pregnancies in which smoking was recorded or the algorithm reclassified mothers as smoking in pregnancy.

^b^ Fewer than 5 pregnancies is the reporting limit for Norwegian and Swedish data.

^c^ In New South Wales, NRT patches were available on subsidy throughout the whole study period, while subsidy for lozenges and gums commenced in late 2019. As this resulted in small cell sizes, no stratification by NRT formulation was performed.

^d^ In Sweden, NRT nasal sprays and inhalers were used by fewer than 5 individuals.

Table 5 describes the timing of use for each smoking cessation pharmacotherapy during pregnancy, along with the patterns of dispensing. Among pregnancies in which varenicline was used, between 89.6% (172 pregnancies in Sweden) and 99.0% (379 pregnancies in New Zealand) of individuals had the medication in their possession sometime during the first trimester; however, 25.5% (49 pregnancies in Sweden) to 47.0% (180 pregnancies in New Zealand) were identified through a single dispensing in the preconception period. Similarly, among pregnancies in which bupropion was used, between 76.9% (20 pregnancies in Sweden) and 89.8% (230 pregnancies in New Zealand) were in possession of the medication during the first trimester. In New Zealand, 90 pregnancies (35.2%) with bupropion were identified through a single dispensing in the preconception period. We were unable to estimate this for other countries due to small cell sizes. Among pregnancies in which prescription NRT was used, between 54.3% (25 pregnancies in Sweden) and 62.0% (4062 pregnancies in New Zealand) were in possession of the medication during the first trimester, with less than 10% identified through a single dispensing in the preconception period. The sensitivity analysis using LMP as the start of the exposure period rather than date of conception yielded similar results as the main analysis (eTable 6 in Supplement 1).

**Table 5.** Timing of Use of Smoking Cessation Pharmacotherapy Use During Pregnancy by Country

Timing of first dispensing in course	Pregnancies, No. (%)			
	New South Wales, Australia	New Zealand	Norway	Sweden
**Varenicline**				
Any varenicline use, No.	643	383	65	192
Preconception and pregnancy use (including first trimester), dispensings				
1	202 (31.4)	180 (47.0)	27 (41.5)	49 (25.5)
≥2	195 (30.3)	65 (17.0)	12 (18.5)	72 (37.5)
Pregnancy use (including first trimester), dispensings				
1	194 (30.2)	105 (27.4)	18 (27.7)	46 (24.0)
≥2	16 (2.5)	29 (7.6)	5 (7.7)	5 (2.6)
Pregnancy use (excluding first trimester), dispensings				
1	28 (4.4)	4 (1.0)^a^	<5^a^	20 (10.4)^a^
≥2	8 (1.2)	NR	NR	NR
Pregnancies with first trimester use^b^	607 (94.4)	379 (99.0)	62 (95.4)	172 (89.6)
**NRT**				
Any NRT use, No.	1062	6551	11	46
Preconception and pregnancy (including first trimester), dispensings				
1	93 (8.8)	478 (7.3)	NA	6 (13.0)^a^
≥2	91 (8.6)	256 (3.9)	<5	NR
Pregnancy (including first trimester), dispensings				
1	388 (36.5)	2945 (45.0)	<5	19 (41.3)^a^
≥2	67 (6.3)	383 (5.8)	NA	NR
Pregnancy (excluding first trimester), dispensings				
1	362 (34.1)	2167 (33.1)	7 (63.6)	21 (45.7)^a^
≥2	61 (5.7)	322 (4.9)	NA	NR
Any first trimester use^b^	639 (60.2)	4062 (62.0)	<5	25 (54.3)
**Bupropion**				
Any bupropion use, No.	43	256	<5	26
Preconception and pregnancy (including first trimester), dispensings				
1	16 (37.2)^a^	90 (35.2)	NR	20 (76.9)^a^
≥2	NR	66 (25.8)	NR	NR
Pregnancy (including first trimester), dispensings				
1	18 (41.9)^a^	55 (21.5)	NR	<5
≥2	NR	19 (7.4)	NR	NA
Pregnancy (excluding first trimester), dispensings				
1	9 (20.9)^a^	14 (5.5)	NR	<5
≥2	NR	12 (4.7)	NR	NA
Any first trimester use^b^	34 (79.1)	230 (89.8)	NR	NR

Abbreviations: NA, not applicable; NR, Not reported due to small cell sizes; NRT, nicotine replacement therapy.

^a^ Includes 2 or more dispensings. Due to small sizes in either category, the reporting limits for each country are fewer than 6 pregnancies for New South Wales, fewer than 3 pregnancies for New Zealand, and fewer than 5 pregnancies for Norway and Sweden data.

^b^ Pregnancies with first trimester use combines the categories of preconception and pregnancy (including first trimester) and pregnancy use (including first trimester).

## Discussion

This cohort study found that the use of varenicline and bupropion in pregnancy was consistently low in the 4 participating countries, with use occurring in only a fraction of a percentage point of all pregnancies. Use was also low in pregnancies with maternal smoking, with maximums of 1.25% for varenicline and 0.39% for bupropion. These low rates of use are reassuring, given the lack of robust safety evidence and the recommendation from some professional bodies to avoid these medications during pregnancy.

In contrast, the prevalence estimates for prescription NRT varied among countries, likely at least partially due to differences in financial incentives for prescription NRT. Prescription NRT was used in almost 2% of pregnancies resulting in birth in New Zealand, while in other countries, these estimates were less than a quarter of a percentage point. Among pregnancies with maternal smoking, the estimates increased to 11% in New Zealand and nearly 2.5% in New South Wales, whereas use estimates in the Nordic countries remained less than a quarter of a percentage point. Although NRT is also available over the counter in New Zealand, the very low out-of-pocket cost for prescription NRT (NZ$5 during the study period [approximately US$3]), including patches, gums, and lozenges, likely results in marginal over-the-counter use. In contrast, in New South Wales, Norway, and Sweden, where NRT is available over the counter as well as via prescription, the out-of-pocket cost for prescriptions is much higher than in New Zealand. This means there is likely to be a nontrivial amount of over-the-counter NRT use in these countries. Cost has been reported as a key component influencing attitudes toward use of NRT during pregnancy, along with factors such as stigma and potential side effects.^32^ All this considered, the New Zealand results are likely to be robust and represent a close estimation of the use of NRT during pregnancy in New Zealand. However, due to differences in population, health systems, and health care professionals’ perceptions toward smoking cessation pharmacotherapies, it may be difficult to generalize these findings to other countries. Our estimates for New South Wales, Norway, and Sweden are likely to be substantial underestimations of the total NRT use during pregnancy and, consequently, prevalence of the use of any smoking cessation pharmacotherapy.

Some guidelines for smoking cessation in pregnancy recommend intermittent NRT formulations, such as lozenges and gums, over transdermal patches because they result in a lower daily dose of nicotine.^14,15^ However, our data from New Zealand show that among pregnancies where NRT was used, patches were used in nearly three-quarters of pregnancies. This accords with qualitative work showing generally negative views on the intermittent forms among pregnant individuals, while there were some positive accounts for using patches.^33^ Additionally, in a randomized clinical trial in which pregnant individuals were given a choice of NRT formulation, 59% of participants chose patches, while 36% of participants chose either gums or lozenges.^34^ Our results indicate that NRT patches were more popular among pregnant individuals;, therefore, it is important for future studies to examine differences in the effectiveness and fetal safety among NRT formulations.

Although smoking cessation, especially in early pregnancy, has several benefits for both maternal and fetal health, these benefits need to be weighed against the potential risks of smoking cessation pharmacotherapies. One critical outcome in assessing pregnancy safety of medications is congenital malformations among infants, an outcome for which the first trimester is the period of vulnerability. Our results indicate that, in most pregnancies in which smoking cessation pharmacotherapies were used, at least some of the use occurred during the first trimester (range, 54.3%-99.0%). These high proportions, especially for varenicline and bupropion, suggest at least some inadvertent use during pregnancy. Even after accounting for potential nonuse among individuals with a single dispensing during the preconception period, a large proportion had some use during the first trimester. This highlights the need to investigate the risk of congenital malformations, especially for NRT, given its higher prevalence of use, and the limited evidence currently available.^28^ A study investigating this risk of congenital malformations is currently underway by our team.

### Strengths and Limitations

The strengths of this study include using whole-population data from 4 countries, analyzed using a common protocol. Our study also has some limitations. Our study population only included pregnancies resulting birth; therefore, our results cannot be applied to pregnancies that ended for any reason prior to 22 weeks’ gestation. Use of smoking cessation pharmacotherapies was determined using dispensing data, which does not necessarily represent actual consumption of the medication. Furthermore, specifically for intermittent forms of NRT, we assumed consumption of 1 defined daily dose for the calculation of days’ supply; however, actual use may vary widely. Since varenicline and bupropion are prescription-only smoking cessation pharmacotherapies, our results are likely to have captured the full use of these medications during pregnancy. Data on over-the-counter NRT purchases were not available; prevalence estimates of NRT use among pregnant individuals in New South Wales, Norway, and Sweden are likely underestimated, as there is no or limited financial incentive to choose prescribed NRT instead of purchasing it over the counter. Furthermore, we had no data on other nicotine products used as smoking cessation aids or for harm reduction, such as electronic cigarettes, snus, and nicotine pouches. It is possible that these products are used to different extents across the jurisdictions participating in this study.

## Conclusions

This cohort study found that very few individuals in New South Wales, Australia; New Zealand; Norway; and Sweden used varenicline or bupropion during pregnancy, consistent with current guidelines. However, a larger proportion of individuals used prescribed NRT at some time during pregnancy. For most pregnancies in which these medications were used, use occurred at least partly during the first trimester of pregnancy, which is the vulnerable period for congenital malformations. Our findings highlight the need for further evidence regarding their safety during pregnancy.

## Notes

Supplement 1. **eTable 1.** Description of Population Data Collection **eTable 2.** Availability and Recommended Dosing of Smoking Cessation Pharmacotherapies in 4 Countries and Days of Supply Calculation **eFigure 1.** Determination of Length of Smoking Cessation Pharmacotherapy Courses **eTable 3.** Application of Smoker Reclassification Algorithm in 4 Jurisdictions **eTable 4.** Definitions of Maternal Characteristics **eTable 5.** Definitions of Morbidities **eFigure 2.** Flow Diagram of Smoker Reclassification Algorithm Based on the Algorithm Developed by Roper et al **eFigure 3.** Cohort Selection Diagram Across New South Wales, Australia; New Zealand; Norway; and Sweden **eTable 6.** Utilization of Smoking Cessation Pharmacotherapies at Any Time During Pregnancy Across 4 Jurisdictions Using Last Menstrual Period as Start of Pregnancy
eReferences.

Supplement 2.
Data Sharing Statement
