# Supplementary material for: Smoking Cessation Pharmacotherapy Use in Pregnancy
Source: JAMA Netw Open. 2024 Jun 28;7(6):e2419245. doi: 10.1001/jamanetworkopen.2024.19245 (PMC11214111; doi:10.1001/jamanetworkopen.2024.19245)
Supplement: Supplement 1. — eTable 1. Description of Population Data Collection eTable 2. Availability and Recommended Dosing of Smoking Cessation Pharmacotherapies in 4 Countries and Days of Supply Calculation eFigure 1. Determination of Length of Smoking Cessation Pharmacotherapy Courses eTable 3. Application of Smoker Reclassification Algorithm in 4 Jurisdictions eTable 4. Definitions of Maternal Characteristics eTable 5. Definitions of Morbidities eFigure 2. Flow Diagram of Smoker Reclassification Algorithm Based on the Algorithm Developed by Roper et al eFigure 3. Cohort Selection Diagram Across New South Wales, Australia; New Zealand; Norway; and Sweden eTable 6. Utilization of Smoking Cessation Pharmacotherapies at Any Time During Pregnancy Across 4 Jurisdictions Using Last Menstrual Period as Start of Pregnancy eReferences. [file jamanetwopen-e2419245-s001.pdf]

Supplemental Online Content

Robijn AL, Tran DT, Cohen JM, et al. Smoking cessation pharmacotherapy use in pregnancy. *JAMA Netw Open*. 2024;7(6):e2419245.
doi:10.1001/jamanetworkopen.2024.19245

eTable 1. Description of Population Data Collection

eTable 2. Availability and Recommended Dosing of Smoking Cessation Pharmacotherapies in 4 Countries and Days of Supply Calculation

eFigure 1. Determination of Length of Smoking Cessation Pharmacotherapy Courses

eTable 3. Application of Smoker Reclassification Algorithm in 4 Jurisdictions

eTable 4. Definitions of Maternal Characteristics

eTable 5. Definitions of Morbidities

eFigure 2. Flow Diagram of Smoker Reclassification Algorithm Based on the Algorithm Developed by Roper et al

eFigure 3. Cohort Selection Diagram Across New South Wales, Australia; New Zealand; Norway; and Sweden

eTable 6. Utilization of Smoking Cessation Pharmacotherapies at Any Time During Pregnancy Across 4 Jurisdictions Using Last Menstrual Period as Start of Pregnancy

eReferences.

This supplemental material has been provided by the authors to give readers additional information about their work.

© 2024 Robijn AL et al. *JAMA Network Open.*

Supplemental methods

Abbreviations

**ACHI**	Australian Classification of Health Interventions
**ATC**	Anatomical Therapeutic Chemical
**BMI**	Body Mass Index
**DoC**	Date of Conception
**ICD-10**	International Statistical Classification of Diseases and Related Health Problems, 10th Revision
**ICD-10-AM**	International Statistical Classification of Diseases and Related Health Problems, 10th Revision-Australian Modification
**MBR**	Medical Birth Register
**NRT**	Nicotine replacement therapy
**NSW**	New South Wales
**NZ**	New Zealand
**PBS**	Pharmaceutical Benefits Scheme (PBS)
**QTY**	Quantity

We conducted a distributed cohort study using linked administrative data from New South Wales (NSW), the most populous state in Australia, New Zealand (NZ), Norway, and Sweden. In each country, whole-of-population data relating to pregnancies resulting in birth were linked to records of prescription medication dispensings, inpatient and outpatient care, and mortality. Specific data sources and the years for which data were available time span coverage in each country are described below.

Table S1. Description of population data collection used in this study.

**Jurisdiction**	**Data collection**	**Description**	**Further resources**
**NSW**	Perinatal Data Collection	Records of all live- and stillbirths of at least 20 weeks gestation or at least 400g birth weight in NSW.	See Tran et al. 2024[1]
	Pharmaceutical Benefits Scheme	Records of every publicly funded pharmaceutical product dispensed in pharmacies or private hospitals	
	Admitted Patient Data Collection	Records of every public and private hospital discharge in NSW	
**New Zealand**	National Maternity Collection	Records of all live- and stillbirths of at least 20 weeks gestation	See Donald et al. 2018[2]
	Pharmaceutical Collection	Records of publicly funded pharmaceutical products dispensed in community pharmacies	
	National Minimum Dataset	Records of all discharges from public hospitals and publicly funded events in private hospitals	
	National Health Index	Demographic information for all people with a National Health Index number.	
	Programme for the Integration of Mental Health Data	The national mental health and addiction information collection of service activity and outcomes data for health consumers.	
**Norway**	Medical Birth Registry of Norway	Records of all live- and stillbirths of at least 12 weeks gestation	See Cohen et Al. 2021,[3] Furu et al. 2010[4]
	Norwegian Prescription Database	Records of all prescribed medications dispensed in pharmacies to individuals	
	Norwegian Patient Registry	Records of all hospital admissions, outpatient consultations, and specialist consultations	

© 2024 Robijn AL et al. *JAMA Network Open.*

**Sweden**	Swedish Medical Birth Register	Records of all live- and stillbirths of at least 22 weeks gestation	See Furu et al. 2010[4], Ludvigson et al. 2011[5], Cnattingius et al. 2023[6]
	Swedish Prescribed Drug Register	Records of all prescription pharmaceutical dispensed in pharmacies	
	Swedish National Patient Register	Records of all hospital admissions, outpatient consultations, and specialist consultations	

New South Wales, Australia

- The NSW Perinatal Data Collection (July 2001 to December 2019) includes records for all live births and stillbirths of at least 20 weeks gestation or at least 400g birth weight delivered in NSW. Information on maternal characteristics, complications during pregnancy and delivery, and neonatal outcomes are recorded by the attending midwife or doctor.
- The Pharmaceutical Benefits Scheme (PBS, January 2001 to December 2020) data include a record of every PBS listed medications dispensed to eligible individuals in community pharmacies and private hospitals. Information included PBS item number, generic name, anatomical therapeutic chemical (ATC) code, date of prescribing, date of supply, quantity, and patient beneficiary status (social security or general). Mediations purchased over the counter and those administered in public hospitals are not included.
- The Admitted Patient Data Collection (July 2001 to June 2020) includes a record for every public and private hospital separation in NSW. Information includes patient demographics, diagnoses, procedures, and external causes of injuries. Diagnoses are coded according to the International Statistical Classification of Diseases and Related Problems, 10th revision – Australian Modification (ICD-10-AM) while procedures are coded according to the Australian Classification of Health Interventions (ACHI).

More details regarding the NSW linked data are reported elsewhere.[1]

New Zealand

- The National Maternity Collection (January 2005-December 2020) is a national collection of pregnancies that resulted in deliveries of live and stillborn infants of at least 20 weeks of gestation, including home births and hospital births. This data collection contains information about pre-pregnancy maternal health, neonatal outcomes, complications during pregnancy, birth and postnatal period for mothers and babies.
- The Pharmaceutical Collection (January 2005-December 2021) contains records of publicly funded pharmaceutical products dispensed in community pharmacies. Individual medications have a unique chemical ID number and are organised according to therapeutic groups. In general, Level 1 therapeutic groups are organised by body system (e.g., nervous system), with Level 2 therapeutic groups organised by functional group (e.g., antidepressants) and Level 3 by medication class (e.g., selective serotonin reuptake inhibitors). Medications purchased over the counter and those administered in public hospitals are not included.
- The National Minimum Dataset (January 1988 – December 2021) is a national collection of public and private hospital discharge information, including coded clinical data for inpatients and day patients. Information includes patient demographics, diagnoses, procedures, and external causes of injuries. Diagnoses are coded according to the ICD-10-AM while procedures are coded according to the ACHI.
- The National Health Index includes demographic data such as ethnicity and an area-based measure of deprivation.
- The Programme for the Integration of Mental Health Data (July 2008 - December 2021) is the national mental health and addiction information collection of service activity and outcomes data for health consumers.

Further details about the New Zealand pregnancy cohort are reported elsewhere.[2]

© 2024 Robijn AL et al. *JAMA Network Open.*

Norway

- The Medical Birth Registry of Norway (January 2004 to December 2020) is a mandatory registration of all live- and stillbirths and pregnancies of at least 12 weeks of gestation. The register contains information about pre-pregnancy maternal health, complications during pregnancy or birth, neonatal outcomes, infant diagnoses, or evidence of congenital abnormalities. It also contains data about miscarriages and pregnancy termination for fetal anomaly.[7]
- The Norwegian Prescription Database (January 2004 to December 2020) is a mandatory registration of all medications dispensed in pharmacies in Norway. Information includes ATC codes, medication strength, defined daily doses in a package, package size and dispensing date. Medications that are purchased over the counter or supplied to hospitals and nursing homes are not included. Since March 2009, indication for reimbursement was coded according to ICD-10 codes and version 2 of the International Classification of Primary Care.[4]
- The Norwegian Patient Registry (January 2008 to December 2020) is a nationwide registry covering all hospital admissions, outpatient consultations, and specialist consultations. Diagnoses (primary and several secondary diagnoses) are coded according to the ICD-10 codes since 2008.[8]

Sweden

- The Swedish Medical Birth Register (January 2005 to December 2019) covers all live- and stillbirths of at least ≥ 22 weeks gestation (gestation ≥28 weeks before 2008). Information included pre-pregnancy maternal health, complications during pregnancy or birth, neonatal outcomes, and infant diagnoses.[6]
- The Swedish Prescribed Drug Register (January 2005 to December 2019) contains complete national data on all prescription pharmaceuticals dispensed. Dispensing records contain ATC codes, date of dispensing, the dose, and quantity. Hospital administered medications, vaccines and over the counter medications are not included.[4]
- The Swedish National Patient Register (January 2005 to December 2019) covers all hospital admissions, outpatient consultations, and specialist consultations. Diagnoses are coded according to ICD-10 codes and surgical procedures coded according to Nordic Classification of Surgical Procedures.[5]

Further information about the data sources of the Nordic Pregnancy Drug Safety Studies collaboration are reported elsewhere.[3,4]

Contextual information regarding smoking cessation pharmacotherapies

Government subsidy schemes for smoking cessation therapies vary between countries.

In Australia, bupropion (subsidised since 2001) and varenicline (subsidised since 2008) are prescription only. NRT products can be purchased over the counter or via prescription. Subsidy for NRT transdermal patches commenced from 2008 for Aboriginal and Torres Strait Islander people, followed by an extension to the general population from 2011.[9] NRT fast-acting formulations were PBS-listed since late 2019,[9] at the end of the period covered by PBS data available to this study.

In New Zealand, nicotine replacement therapies (NRT) such as patches, lozenges, and gums, bupropion and varenicline were subsidised from at least 2004 (exact date unknown), 2009 and 2010 respectively. Although NRT products can be purchased over the counter, discussions with New Zealand tobacco control experts,

© 2024 Robijn AL et al. *JAMA Network Open.*

pharmacists and Pharmac (the New Zealand medication buying agency) suggest that most of the NRT obtained by individuals is likely to be via prescription and other sources captured by Pharmaceutical Collection data (author LP personal communications).

In Norway, varenicline is available through prescription only (reimbursed since 2007). NRT products are mostly available over the counter (since 2005), although preliminary analyses found small number of records of prescription NRT were filled during pregnancy. Bupropion is licensed for depression treatment only, the sustained-release bupropion (sold under brand name Zyban™) is indicated for smoking cessation. Zyban™ 150mg was marketed and dispensed (with the indication smoking cessation) from 2000 in Norway.

In Sweden, varenicline is available through prescription only (reimbursed since 2007). NRT patches and lozenges are available mostly over the counter (since 2006). Bupropion is licensed for depression treatment only, the sustained-release bupropion (sold under brand name Zyban™) is indicated for smoking cessation.

Table S2 shows the availability, recommended dosing, and recommended full course across the four jurisdictions, and calculation of days of supply for each dispensing item. In Australia, Norway and Sweden, medications are coded according to the ATC classification system while in New Zealand, the chemical ID (ChemID) is used.

Table S2. Availability and recommended dosing of smoking cessation pharmacotherapies across four countries and days of supply calculation.

Medications/ code	Form	Strength	Pack size	Defined Daily Dose (DDD) and recommended full course	Quantity	Days' supply
**Varenicline**						
**ATC N07BA03**<br>or<br>**NZ ChemID 3920**	Tablet	0.5 and 1mg	25	0.5mg once a day for days 1-3, 2x 0.5mg per day for days 4-7, 2x 1mg per day for remainder of treatment.<br><br>Full course = 12 weeks (84 days)	QTY = pack size x number dispensed	14
	Tablet	0.5 and 1mg	53			28
	Tablet	1mg	56 or 112			QTY/2
	Tablet	0.5 and 1mg	165			84
	Tablet	0.5 and 1mg	53			28
	Tablet	1mg	56			QTY/2
**Bupropion**						
**ATC N07BA02/N06AX12**<br>or<br>**NZ ChemID 3892**	Tablet	150mg	30/90	2x 150mg per day.<br><br>Full course = at least 7 weeks (49 days)	QTY = pack size x number dispensed	QTY/2
**Nicotine Replacement Therapy (NRT)**						
**ATC N07BA01**<br>or<br>**NZ ChemID 3722**	Patch	7/14/21/25* mg	1/7/28	One patch per day<br><br>Full course = 8-12 weeks (56-84 days)	QTY = pack size x number dispensed	QTY/1
	Lozenge	1/2/4[#] mg per piece	36/96/216/384	As needed, maximum of 30mg per day<br><br>Full course = 8-12 weeks (56-84 days)	QTY=strength x pack size x number dispensed	QTY/30
	Gum	1[¥]/2/4 mg per piece	96/216/384			
	Spray[§]	13.6mg/ml	13.2ml			
	Inhaler[§]	15mg	20	As needed, maximum of 60mg per day		QTY/60

© 2024 Robijn AL et al. *JAMA Network Open.*

Medications/ code	Form	Strength	Pack size	Defined Daily Dose (DDD) and recommended full course	Quantity	Days' supply
				Full course = 8-12 weeks (56-84 days)		
*25mg patch not available on subsidy in New Zealand;<br>#4mg lozenge not available on subsidy in New Zealand;<br>¥1mg gum not available on subsidy in New Zealand;<br>§Spray and Inhaler not available on subsidy in New Zealand						

© 2024 Robijn AL et al. *JAMA Network Open.*

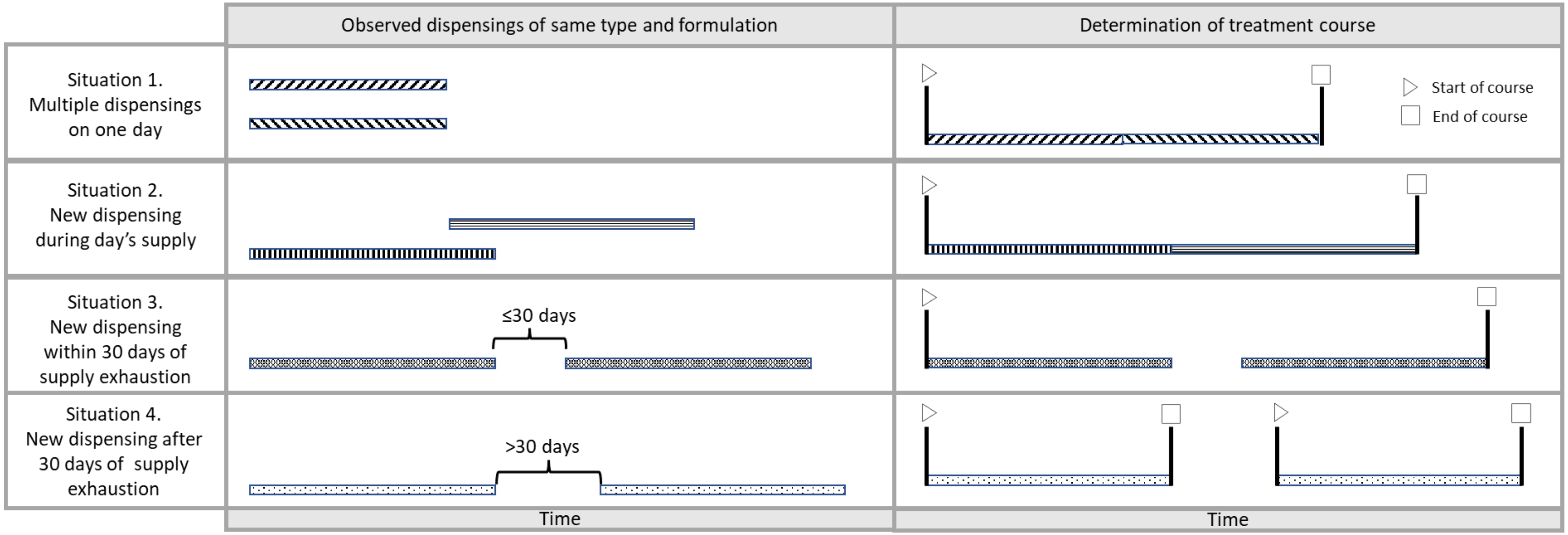

FigureS1. Determination of length of smoking cessation pharmacotherapy courses, start of bar indicates date of dispensing and the length of the bar represents the days of supply of the dispensing calculated based on recommended daily dose (Table S2).

In order to define a course of treatment, we first selected all dispensing records that occurred between 200 days before DoC and date of childbirth. For each dispensing, we calculated days of supply based on amount dispensed and recommended daily dose (see supplemental Table S2). When there were multiple dispensing records, we summed up the days of supply, allowing for a 30-day gap between supply exhaustion and the subsequent dispensing. We considered a woman having another course of treatment when there was a gap >30 days between dispensings (see supplemental Figure 1). Only courses overlapping with the gestation period were included in the analyses.

© 2024 Robijn AL et al. *JAMA Network Open.*

Table S3. Application of smoker re-classification algorithm (Figure S2) across four jurisdictions.

		NSW, Australia		New Zealand		Norway		Sweden	
**Step**	Box	Courses (n)	Births (n)	Courses (n)	Births (n)	Courses (n)	Births (n)	Courses (n)	Births (n)
**1**	Use of SCP	1,870	1,728	10,991	7,100	91	77	326	261
**2**	Mothers NOT recorded	593	559	2,755	1,899	46	39	179	145
**2**	Mother recorded as smoking	1,277	1,169	8,236	5,201	45	38	147	116
**3**	Dispensing PRIOR	269	-	527	-	29	-	106	-
**3**	Dispensing AFTER	324	-	2,228	-	17	-	73	-
**4**	Duration GREATER OR EQUAL	161	-	351	-	10	-	80	-
**4**	Duration LESS	108	-	176	-	36	-	99	-
**5**	MPR ≥ 80%	149	-	271	-	44	-	176	-
**5**	MPR < 80%	12	-	80	-	2	-	3	-
**6**	NO smoking indicated	149	149	271	261	7	7	66	65
**6**	Smoking indicated	444	416	2,484	1,695	39	32	113	80
**7**	NOT SMOKING	-	143	-	204	-	6	-	51
**7**	RE-CLASSIFIED as smoking	-	416	-	1,695	-	33	-	94

© 2024 Robijn AL et al. *JAMA Network Open.*

Table S4. Definitions of maternal characteristics

Maternal characteristics	Jurisdiction-specific definition
Calendar year of childbirth	• Based on date of childbirth
Maternal age at delivery	• As recorded in perinatal/maternity data/ medical birth registries
Indigenous status	• Australia: Aboriginal and/or Torres Strait Islander status recorded in the most recent perinatal record; if missing supplemented by information recorded in the corresponding delivery hospital admission record<br>• New Zealand: Māori ethnicity ever recorded in perinatal, hospital admission, mortality, National Health Index or pharmaceutical dispensing records.<br>• Norway and Sweden: Not applicable
Country of birth	• Australia: Country of birth recorded in the most recent perinatal record, if missing, supplemented by information recorded in the hospital admission corresponding to the childbirth delivery<br>• New Zealand: Not available<br>• Norway/Sweden: As recorded in MBR or population register
Socio-economic disadvantage of residential area	• Australia: Geocoded statistical area, recorded in hospital delivery admission and mapped to ABS 2016 Census, SEIFA Relative Socio-economic Disadvantage (IRSD)<br>• New Zealand: The NZ Index of Deprivation (NZDep)<br>• Norway/Sweden: not available
Remoteness of residence	• Australia: based on the Australian Accessibility and Remoteness Index of Australia Plus, mapped to the area of maternal residence. There are five categories: major cities, inner regional, outer regional, remote and very remote.<br>• New Zealand: based on the Geographical Classification for Health which is similar to the Australian Accessibility and Remoteness Index of Australia Plus. There are five categories: Urban 1, Urban 2, Rural 1, Rural 2, and Rural 3<br>• Norway/Sweden: not available
Body Mass Index (BMI) in early pregnancy	• Australia: weight and height measures available since 2016, may contain missing data<br>• New Zealand: BMI is recorded in the National Maternity Collection (some missing data)<br>• Norway/Sweden: As recorded in MBR or calculated from maternal weight and height at first antenatal visit. May contain missing data
Smoking	As recorded in the perinatal data collections<br>• Australia: smoked in first 20 weeks' gestation<br>• New Zealand: smoking at lead maternity carer registration<br>• Norway: smoking in first trimester<br>• Sweden: smoking in first trimester (reported at first antenatal visit)
Quantity smoked daily	As recorded in the perinatal data collections<br>• Australia: amount smoked in first 20 weeks' gestation

© 2024 Robijn AL et al. *JAMA Network Open.*

Maternal characteristics	Jurisdiction-specific definition
	• New Zealand: amount smoked at first antenatal visit<br>• Norway: amount smoked in first trimester<br>• Sweden: amount smoked at first antenatal visit
Parity	• As recorded in perinatal, maternity data, medical birth registries
Pre-existing maternal morbidities	Table S4

Table S5. Definitions of morbidities

Maternal morbidity	In-hospital data, perinatal data, and other sources	Dispensing data ATC codes * (Pratt et al. 2017[10] Rx Risk, NSW data)
	**Lookback period for ascertainment: DoC-365 to date of delivery, (unless indicated otherwise)**	**Lookback period for ascertainment: DoC-365 to DoC-1**
Mental health disorder	ICD-10 codes: F31-F34, F38, F39, F40, F41, F44, F48, F20-F25, F28-F30, O99.3,<br><br>For NZ, we additionally included the national mental health and addiction service activity and outcomes data	N05BA01 - N05BA12, N05BE01<br>N05AN01**<br>N06AA01-N06AG02, N06AX03 - N06AX11, N06AX12, N06AX13 - N06AX18, N06AX21 - N06AX26,<br>N05AA01 - N05AB02, N05AB06 - N05AL07, N05AX07 - N05AX13
Chronic airways disorder	ICD-10 codes: J31, J32, J35, J37, J40-J44, J47, R05, O99.5<br><br>**(DoC-365 to DoC-1):** ICD-10 codes: J45, J46, J98, J99,<br>Norway: Check box for asthma [£]	R03AC02 - R03DC03, R03DX05
Gastro-oesophageal reflux (GORD)	**(DoC-365 to DoC-1):** K21.0, K21.9	A02BA01 - A02BX05
Thyroid disorder	ICD-10 codes: E00-E07, E89.0	H03BA02 - H03BB01,H03AA01 - H03AA02

© 2024 Robijn AL et al. *JAMA Network Open.*

Maternal morbidity	In-hospital data, perinatal data, and other sources	Dispensing data ATC codes * (Pratt et al. 2017[10] Rx Risk, NSW data)
	**Lookback period for ascertainment: DoC-365 to date of delivery, (unless indicated otherwise)**	**Lookback period for ascertainment: DoC-365 to DoC-1**
Pre-existing diabetes	ICD-10 codes: E10-E14, O24.0, O24.1, O24.2, O24.3<br>NSW perinatal data: a check-box for pre-existing diabetes £<br>Norway: Check box for pre-existing diabetes £	A10A<br>A10BB – A10BK§§
Pre-existing hypertension	ICD-10 codes: I10, I11, I12, I13, I15, O10<br>NSW perinatal data: a check-box for chronic hypertension £<br>Norway: Checkbox for hypertension £	C03AA01–C03BA11, C03DB01, C03DB99,C03EA01, C09BA02– C09BA09, C09DA01, C09DA02–C09DA08, C02AB01–C02AC05, C02DB02–C02DB99,(C09AA01- C09AX99 or C09CA01–C09CX99)‡‡,
Epilepsy	ICD-10 codes: G40, F80.3<br>Norway: Checkbox for epilepsy £	N03AA01 - N03AX99<br>Norway / Sweden: did not include this criteria since the indications for N03A are broader than epilepsy

*: NZ Chemical IDs were mapped to these ATC codes.
** Lithium (WHO N05AN01 code) was recorded as N06AX in PBS data
£: Perinatal record supplements the identification of pre-existing diabetes, pre-existing hypertension, epilepsy and asthma if these conditions were not recorded in hospital records.
‡‡: Either (C09AA01- C09AX99) or (C09CA01–C09CX99) but not both.
§§ Metformin (ATC A10BA) was not included due to its use for other conditions among women of reproductive age, such as polycystic ovary syndrome.

© 2024 Robijn AL et al. *JAMA Network Open.*

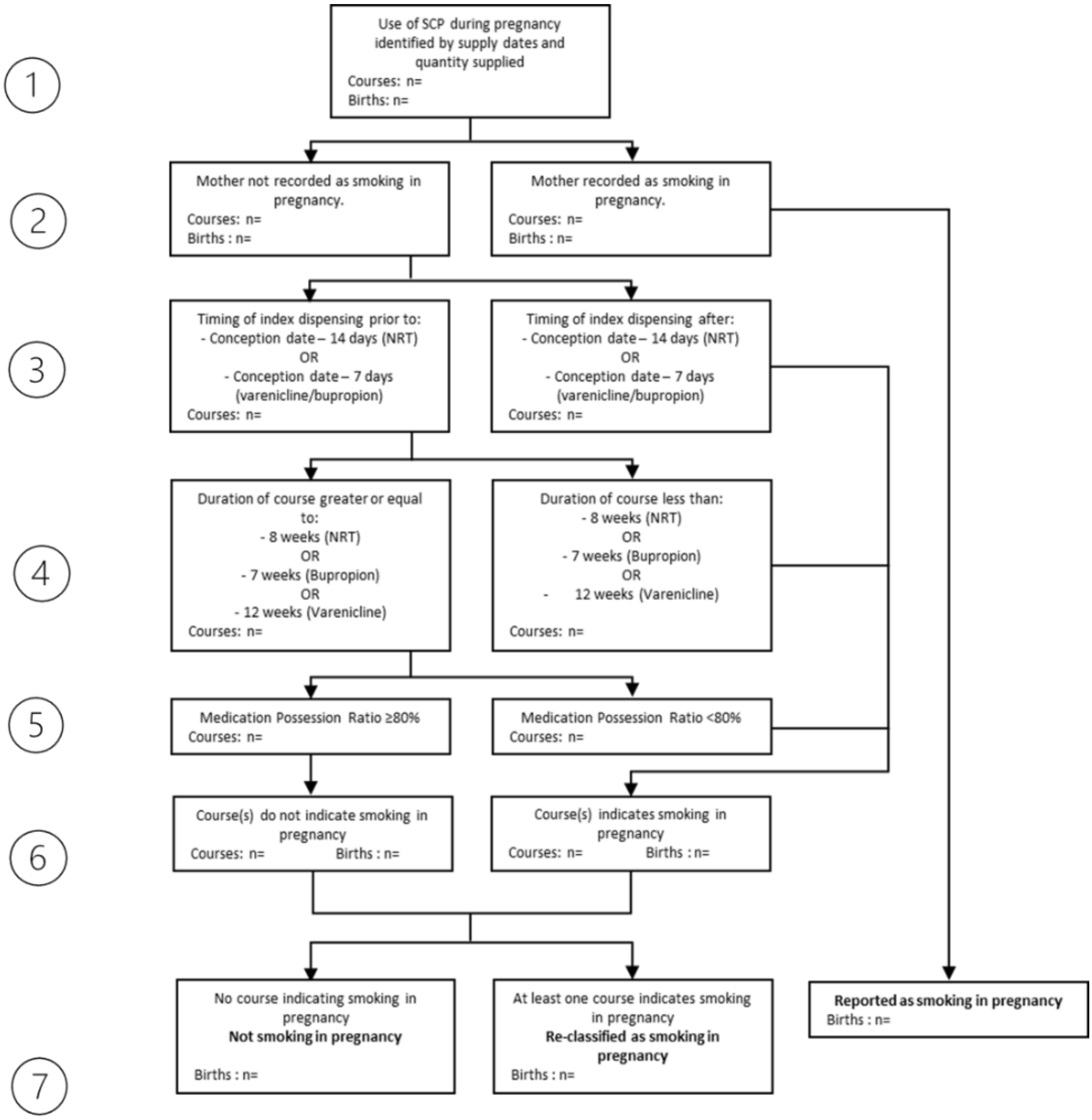

© 2024 Robijn AL et al. *JAMA Network Open.*

Figure S2. Flow diagram of smoker re-classification algorithm based on the algorithm developed by Roper et al. PLOS ONE 2018.[11] Algorithm has been amended to account for pregnancies with multiple courses of smoking cessation pharmacotherapy which could be represented by both sides of the algorithm, addition of step 6 allows us to re-classify all pregnancies with at least one indication of smoking.

Supplemental results

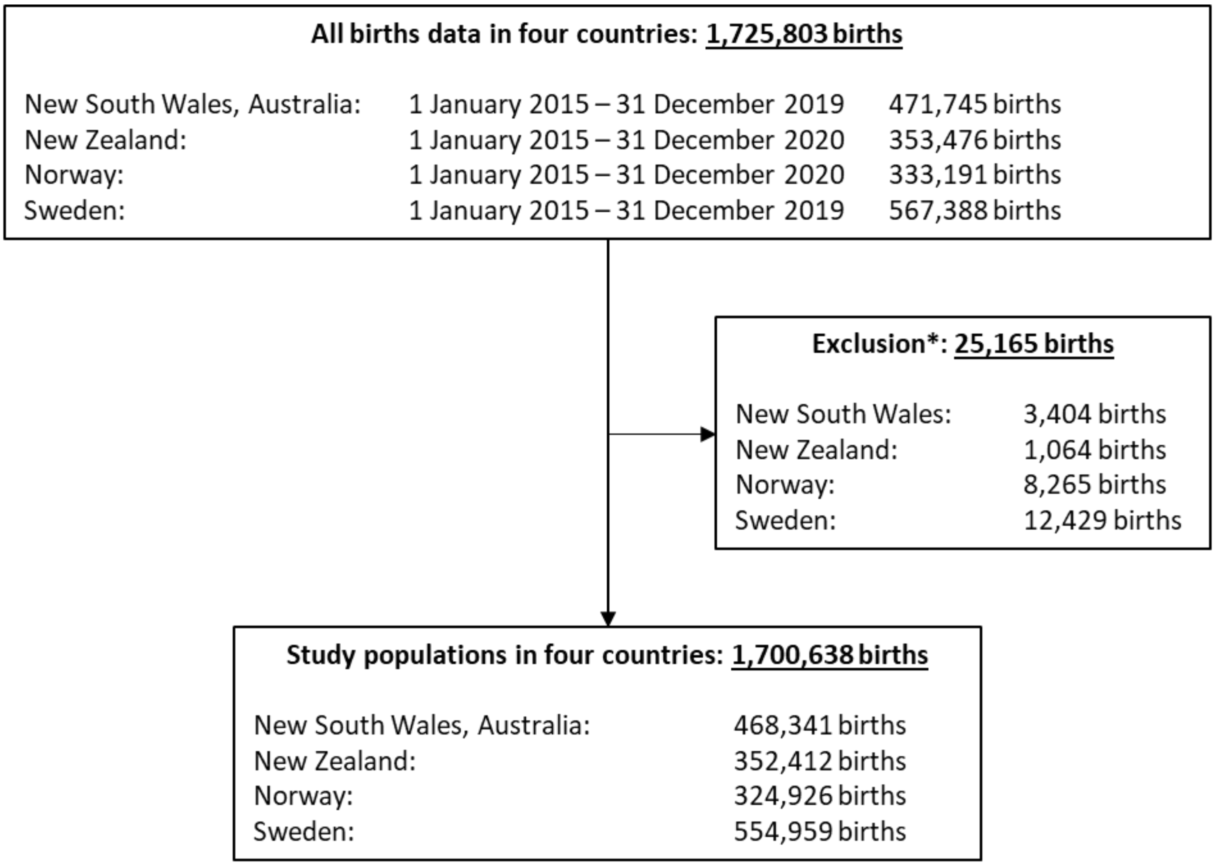

* Exclusion based on missing or invalid gestation age (n=2,512) or overseas visitors/immigration during pregnancy (n=22,650)

Figure S3. Cohort selection diagram across New South Wales (NSW) Australia, New Zealand, Norway, and Sweden.

Table S6. Utilisation of smoking cessation pharmacotherapies at any time during pregnancy across four jurisdictions using last menstrual period as start of pregnancy.

	NSW	NZ	NOR	SWE
All births	N=468,341	N=352,412	N=324,926	N=554,959
Any smoking cessation pharmacotherapy	1,906 (0·41)	7,368 (2·1)	99 (0·03)	292 (0·05)
Varenicline	774 (0·17)	444 (0·13)	85 (0.03)	216 (0·04)
Bupropion	49 (0·01)	303 (0·09)	NR	30 (0·01)
Any Prescription NRT	1,112 (0·24)	6,731 (1·9)	NR	49 (0·01)
Patches		4,992 (1·4)		28 (0·01)
Lozenges		2,159 (0·6)		7 (<0·01)
Gums		2,700 (0·8)		10 (<0·01)
Nasal spray		N/A		<5
Inhaler		N/A		<5

eReferences

1. Tran DT, Robijn AL, Varney B, et al. Data resource profile: The Early Life Course data platform for research on perinatal and early childhood exposures and outcomes in Australia. *Int J Epidemiol*. 2024;(accepted 7Feb2024).

2. Donald S, Barson D, Horsburgh S, Sharples K, Parkin L. Generation of a pregnancy cohort for medicine utilisation and medicine safety studies in New Zealand. *Pharmacoepidemiol Drug Saf*. 2018;27(12):1335-1343. doi:10.1002/pds.4671

3. Cohen JM, Cesta CE, Kjerpeseth L, et al. A common data model for harmonization in the Nordic Pregnancy Drug Safety Studies (NorPreSS). *Norsk Epidemiologi*. 2021;29(1-2):117-123. doi:10.5324/nje.v29i1-2.4053

4. Furu K, Wettermark B, Andersen M, Martikainen JE, Almarsdottir AB, Sørensen HT. The Nordic countries as a cohort for pharmacoepidemiological research. *Basic Clin Pharmacol Toxicol*. 2010;106(2). doi:10.1111/j.1742-7843.2009.00494.x

5. Ludvigsson JF, Andersson E, Ekbom A, et al. External review and validation of the Swedish national inpatient register. *BMC Public Health*. 2011;11. doi:10.1186/1471-2458-11-450

6. Cnattingius S, Källén K, Sandström A, et al. The Swedish medical birth register during five decades: documentation of the content and quality of the register. *Eur J Epidemiol*. 2023;38(1):109-120. doi:10.1007/s10654-022-00947-5

7. Irgens LM. The Medical Birth Registry of Norway. Epidemiological research and surveillance throughout 30 years. *Acta Obstet Gynecol Scand*. 2000;79(6). doi:10.1034/j.1600-0412.2000.079006435.x

© 2024 Robijn AL et al. *JAMA Network Open.*

8. Ludvigsson JF, Håberg SE, Knudsen GP, et al. Ethical aspects of registry-based research in the Nordic countries. *Clin Epidemiol*. 2015;7.

9. Pharmaceutical Benefits Scheme. *Post-Market Review of Medicines for Smoking Cessation – Final Report to the PBAC.*; 2022.

10. Pratt NL, Kerr M, Barratt JD, et al. The validity of the Rx-Risk Comorbidity Index using medicines mapped to the Anatomical Therapeutic Chemical (ATC) Classification System. *BMJ Open*. 2018;8(4):e021122. doi:10.1136/bmjopen-2017-021122

11. Roper L, Tran DT, Einarsdóttir K, Preen DB, Havard A. Algorithm for resolving discrepancies between claims for smoking cessation pharmacotherapies during pregnancy and smoking status in delivery records: The impact on estimates of utilisation. *PLoS One*. 2018;13(8):e0202999. doi:10.1371/journal.pone.0202999

© 2024 Robijn AL et al. *JAMA Network Open.*
