# Supplementary material for: Smoking Cessation Pharmacotherapy Use in Pregnancy
Source: JAMA Netw Open. 2024 Jun 28;7(6):e2419245. doi: 10.1001/jamanetworkopen.2024.19245 (PMC11214111; doi:10.1001/jamanetworkopen.2024.19245)
Supplement: Supplement 2. — Data Sharing Statement [file jamanetwopen-e2419245-s002.pdf]

Data Sharing Statement

Robijn. Smoking Cessation Pharmacotherapy Use in Pregnancy. *JAMA Netw Open*. Published June 28, 2024. doi:10.1001/jamanetworkopen.2024.19245

Data
Data available: No

Additional Information
Explanation for why data not available: The data underlying this article cannot be shared publicly due to agreements and terms under current ethics approvals.
